# Visualizing Academic Contributions to Achieving the Sustainable Development Goals through AI: The Case of Universitat Politècnica de València

**DOI:** 10.1021/acssusresmgt.4c00074

**Published:** 2024-04-03

**Authors:** Débora Domingo-Calabuig, Sergio Hoyas, Ricardo Vinuesa, J. Alberto Conejero

**Affiliations:** † Dpto. Proyectos Arquitectónicos, Universitat Politècnica de València, València 46022, Spain; ‡ Instituto Universitario de Matemática Pura y Aplicada, Universitat Politècnica de València, València 46022, Spain; ¶ FLOW, Engineering Mechanics, KTH Royal Institute of Technology, Stockholm 114 28, Sweden; § KTH Climate Action Centre, Stockholm, SE-100 44, Sweden; ∥ Instituto Universitario de Matemática Pura y Aplicada, Universitat Politècnica de València, València 46022, Spain

**Keywords:** Sustainable development goals, artificial intelligence, language models, scientific publications

## Abstract

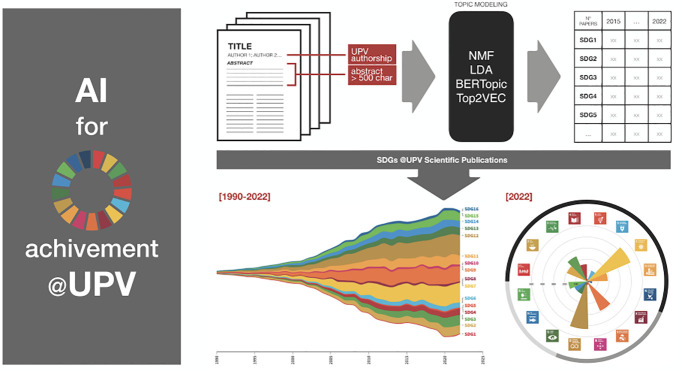

How are you contributing to SDGs and measuring sustainable improvements? AI solutions can help you to quantify it. This pilot experience shows the case of the university’s scientific contributions.

Spanish universities face the challenge of the ecological transition holistically and comprehensively. Regardless of the initiatives that each institution may have undertaken previously, two recent national laws emphasize the need to demonstrate progress in the sustainability of universities through compliance with requirements and the development of plans that justify their resource management: the Climate Change and Energy Transition Law of 2021^1^ and the Organic Law of the University System of 2023.^2^ Thus, university budgets, undergraduate and master’s curricula, academic reports, organization of events, and scientific production must be held accountable for environmental, economic, and social sustainability.

The Universitat Politècnica de València (UPV) in Spain is highly committed to sustainability, as demonstrated by several recent achievements. It is the only Spanish public university with the European EMAS (Eco-Management and Audit Scheme) seal;^3^ it has the lowest carbon footprint per person of all Spanish higher-education institutions and occupies relevant positions in UI GreenMetrics World University Ranking,^4^ Sustainability QS World University Rankings,^5^ and The Times Higher Education Ranking.^6^ The commitment to legislative compliance transcends diverse actions, such as the recognition in its 2023–2027 strategic plan of sustainability as the first pillar of the institution or the integration of the Sustainable Development Goals (SDGs) in the preparation of its budget lines. However, given the diversity of actions undertaken in a university with more than 30,000 students, more than 3000 staff members, and 41 degrees (data from 2022), it is necessary to establish a system capable of verifying sustainable management and progress in achieving the SDGs.

Since 2017, UPV has developed a yearly SDG data monitoring report. Each report addresses each SDG individually, adapting them to the university goals and to the strategic and monitoring indicators. The latter provides a quantitative analysis that is essential for further action. It is completed with more profound knowledge provided by the university units in charge of resource management, mainly the Environment Unit and the Infrastructure Service, ascribed to the Vice-Rectorate Office for Sustainable Development of Campus. For example, the *SDG11 - Sustainable Cities and Communities* report refers to the strategic plan for sustainable mobility, among other initiatives and programs. The monitoring indicator is the *percentage of sustainable means of transport used by the university*, a value obtained from data extracted from the different mobility diagnoses. The report also includes those university degrees that train students in sustainable transportation and the research centers at UPV that produce notable advances in the subject. Similarly, the *SDG15 - Life on Land* report gathers the general programs and actions related to the sustainable environmental management and biodiversity protection of university green spaces. However, greater details can be demonstrated when initiatives like the campus biodiversity inventory are disseminated. As in the previous case, the university also contributes to *SDG15* by training future environmental engineers or transferring knowledge to society through the scientific production of specialized R&D groups.

These examples show the diversity of data needed in managing environmental resources to improve decision making and how complex it is to model indicators to validate progress toward a specific SDG. A solution based on artificial intelligence (AI) that can link texts to the scope of each SDG has been developed to cover this gap. AI has shown to be an essential tool to advance the SDG’s agenda despite its risks and trade-offs.^7−10^ Furthermore, the recent impact of large language models (LLM) is yet to be fully explored for advancing climate research^11,12^ and maintaining our human activity within the planetary boundaries.^13,14^ Furthermore, applying AI methods should also be aligned with the sustainability principles.^15^

As a first step, we have an initial pilot experience with this AI solution to quantify the impact of the scientific contributions of the university members to the SDGs agenda. For this experience, we have downloaded 50,000 abstracts of works with at least one author from UPV and more than 500 characters in length since 1990. Our solution is based on the Automatic Classification of Impact to Sustainable Development Goals (ASDG) pipeline,^16^ which relies on four language models (NMF, LDA, BERTopic, and Top2VEC). This has been applied to automatically identify these scientific contributions’ potential impact on the SDG agenda. Figure 1 shows some graphical analysis from these publications downloaded from Scopus. On the left, we have seen how the number of publications has increased over time (A) and the relative importance of contributions to SDGs. The technological profile of UPV yields that the most relevant contributions are linked to *SDG7 - affordable and clean energy*, *SDG9 - industry, innovation, and infrastructure*, and *SDG12 - responsible production and consumption*. It is also worth mentioning that the potential impact is very limited for *SDG1 - no poverty*, *SDG5 - gender equality*, *SDG8 - decent work and economic growth*, and *SDG10 - reduce inequalities*. To better visualize the distribution of SDG publications, we have represented a normalized distribution of publications per SDG (B). This second graph better illustrates the evolution along the time of the university profile. A more precise representation of the publication’s contribution across SDGs and in terms of SDG Profile (Society, Economy, Environment) through a radial plot is also provided (C).

**Figure 1:**
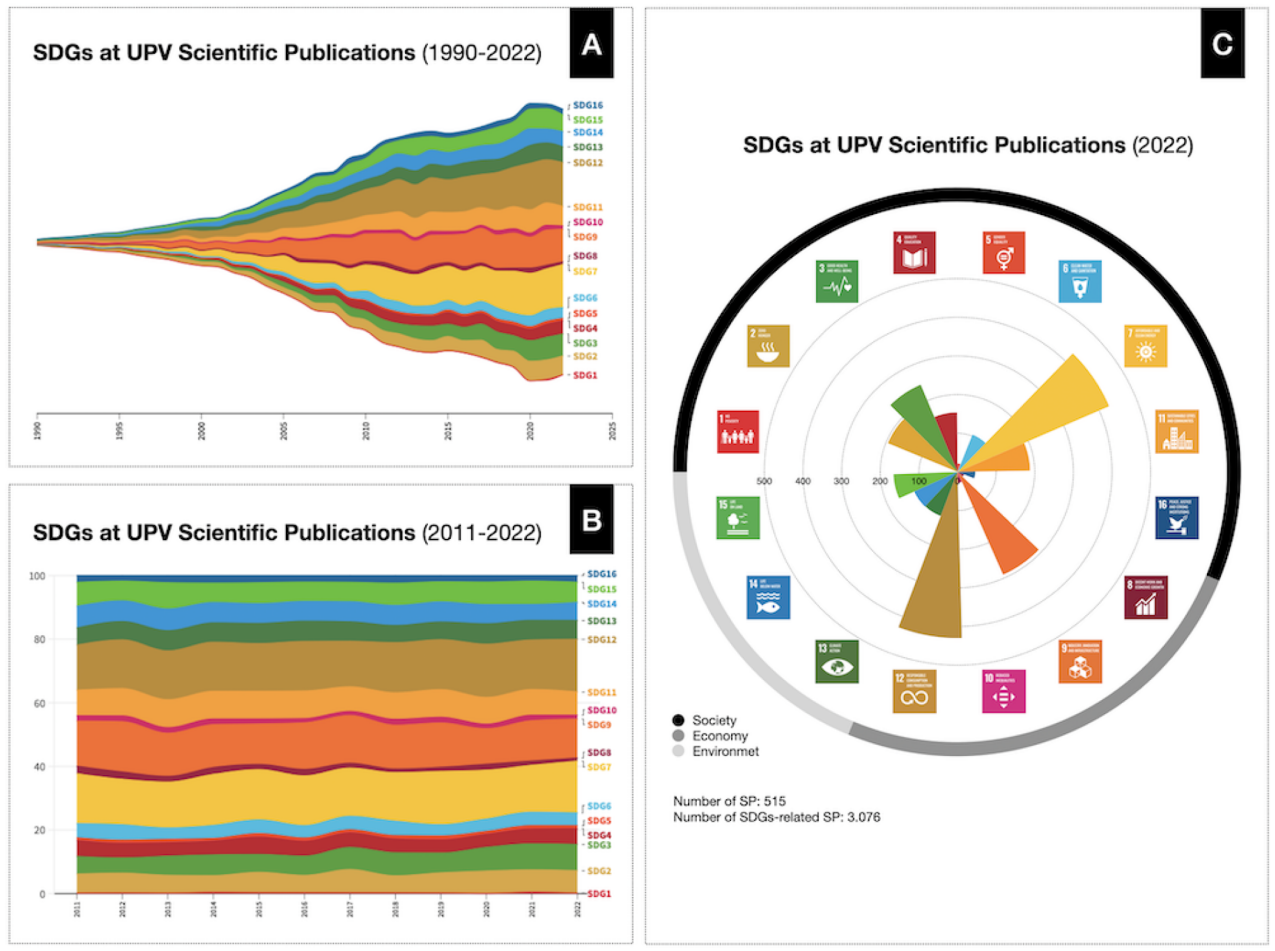
(A) Evolution of the amount of publications linked to the scope of each SDG (1990-2022). (B) Relative distribution of publications across the SDGs (2011–2022). (C) Distribution of publications from 2022 across Society, Economic, and Environmental SDGs.

Currently, this initiative is in the development phase and serves to set up strategic indicators on scientific production to evaluate its alignment with the 2030 agenda. It also allows one to establish comparisons with other Spanish and European universities. Nevertheless, our objective is to expand its application field to all documentary spheres of the university. Academic syllabi and works, or financial and management reports, are texts stored and archived on university servers that could be examined from the sustainability point of view. The tool would aid in understanding the university’s historical documentation and would be helpful in understanding more clearly the contribution of scientific knowledge to SDGs. Furthermore, it could also be applied to implement sustainable management policies and verify their compliance.
